# COVID-19-Induced Bile Duct Casts and Cholangitis: A Case Report

**DOI:** 10.7759/cureus.14560

**Published:** 2021-04-19

**Authors:** David Sanders, Shivanand Bomman, Shayan Irani

**Affiliations:** 1 Gastroenterology, Virginia Mason Medical Center, Seattle, USA

**Keywords:** covid 19, cholangitis, ercp, endoscopic retrograde cholangiopancreatography (ercp)

## Abstract

Coronavirus disease 2019 (COVID-19) infection can lead to various complications involving all of the major organ systems. Gastrointestinal manifestations such as nausea, vomiting, and diarrhea are commonly associated with this condition. Biliary complications from COVID-19 constitute an area of active research. In this report, we present a case of secondary sclerosing cholangitis in a critically ill patient (SSC-CIP) associated with COVID-19. A 57-year-old male with a past medical history of hypertension and diabetes presented to the hospital with signs of sepsis. He had abdominal pain, fever, and elevated liver enzymes without an elevated lipase. Abdominal ultrasound and CT scan showed a dilated common bile duct (CBD) with a distal CBD stone. He had experienced a prolonged course of severe critical illness related to COVID-19 prior to this episode, with respiratory failure requiring mechanical ventilation, thromboembolic complications, and he had also required tracheostomy and gastrostomy tube. The patient was diagnosed with cholangitis and was appropriately treated with antibiotics and fluid resuscitation. An endoscopic retrograde cholangiopancreatography (ERCP) was performed. During ERCP, the biliary cast was removed and a bile duct stent was placed. After the procedure, he showed significant improvement and was discharged on an appropriate course of antibiotics. Outpatient ERCP was eventually done to remove the stent and further bile duct casts were removed. The patient was referred for outpatient cholecystectomy.

Critical illness due to COVID-19 can result in SSC-CIP. This can be further complicated by bile duct casts, liver fibrosis, and cirrhosis.

## Introduction

Coronavirus disease 2019 (COVID-19) can often be asymptomatic, but symptomatic infections result in fever, fatigue, dyspnea, pneumonia, and acute respiratory distress syndrome [1]. Gastrointestinal symptoms are also common and these include anorexia, nausea, vomiting, and diarrhea [2]. COVID-19-associated liver injury with elevated liver enzymes has also been well described in the literature [3]. However, the interaction between COVID-19 and cholelithiasis and its complications is not well understood. Critical illness can cause secondary sclerosing cholangitis in critically ill patients (SSC-CIP), which can lead to bile duct casts [4]. This case report describes the disease course of a male patient with severe COVID-19, and his progression to SSC-CIP and cholangitis secondary to biliary obstruction from bile duct casts.

## Case presentation

A 57-year-old male with hypertension and diabetes presented to an outside hospital with rigors and fatigue in February 2021. His past medical history was notable for a prolonged hospitalization with COVID-19 in November of 2020. Prior to that admission, he had had a good performance status and was not known to have any intrinsic liver disease. Unfortunately, he had developed severe COVID-19 pneumonia, respiratory failure, and had received a tracheostomy due to an inability to wean off the ventilator. His course was further complicated by a right atrial and right ventricular thrombus with pulmonary embolisms. He was managed with a percutaneous endoscopic gastrostomy (PEG) tube and was discharged from a skilled nursing facility three months after the admission. He was not known to have complicated biliary disease. He had not experienced any previous episodes of cholangitis, pancreatitis, or obstructive jaundice. His imaging during his hospitalization did not identify any liver disease. 

**Figure 1:**
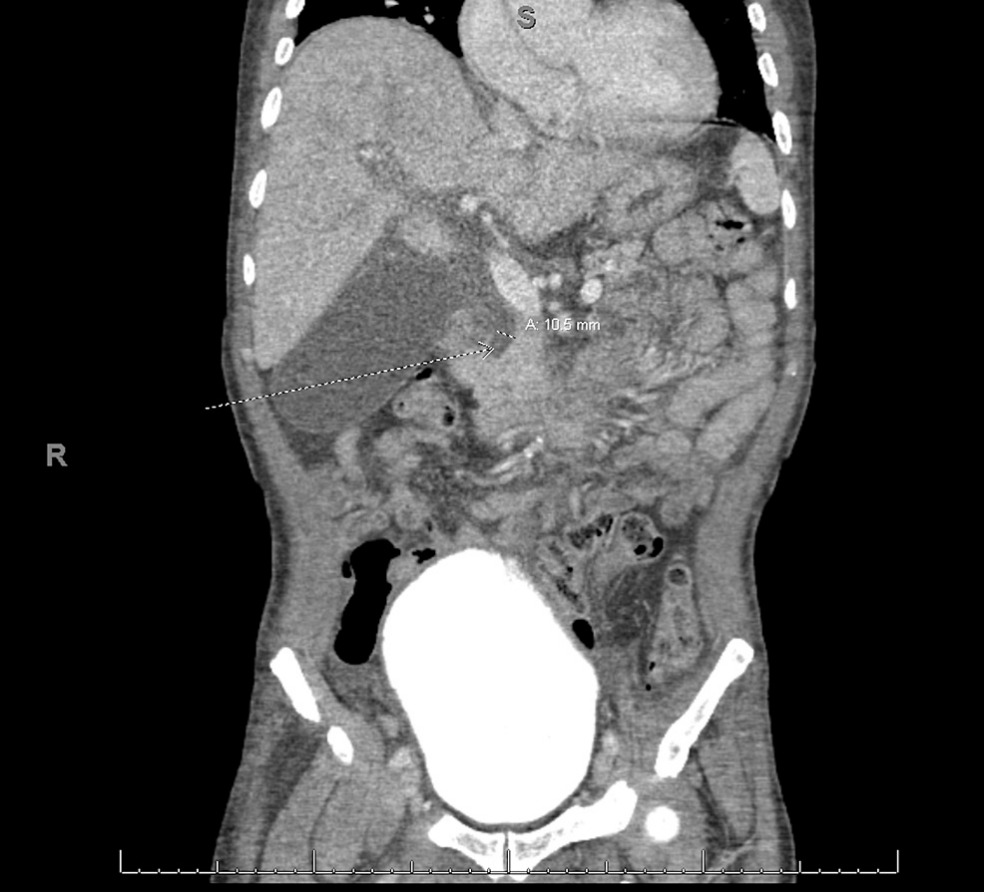
CT showing distal bile duct stones and extrahepatic ductal dilation (10.5 mm) CT: computed tomography

He was brought back to a medical facility one month after his discharge. He was found to have a fever of 38.3 °C, tachycardia, and hypotension. His initial blood work showed leukocytosis with a WBC count of 19 x 10^9^/L, alkaline phosphatase of 870 U/L (normal range: 30-135 U/L), alanine aminotransferase (ALT) of 77 U/L (normal range: 10-55 U/L), and bilirubin of 1.3 mg/dL. His ultrasound showed cholelithiasis and a dilated common bile duct (CBD) (9 mm). An echogenic focus consistent with a stone was noted in the distal CBD on ultrasound. Repeat COVID-19 polymerase chain reaction (PCR) testing was negative twice. He was diagnosed with cholangitis and was resuscitated with 5 L of crystalloids and broad-spectrum antibiotic coverage. He progressed to septic shock and was stabilized on norepinephrine with improvement in his serum lactate. He was transferred to our institution for a higher level of care and consideration of performing an endoscopic retrograde cholangiopancreatography (ERCP). His CT scan upon arrival showed a moderately distended gallbladder. There was mild gallbladder wall thickening and pericholecystic fluid consistent with acute cholecystitis. In line with the ultrasound findings, several tiny stones in the distal CBD were found (Figure 1). His anticoagulation was held for 24 hours prior to his ERCP.

**Figure 2:**
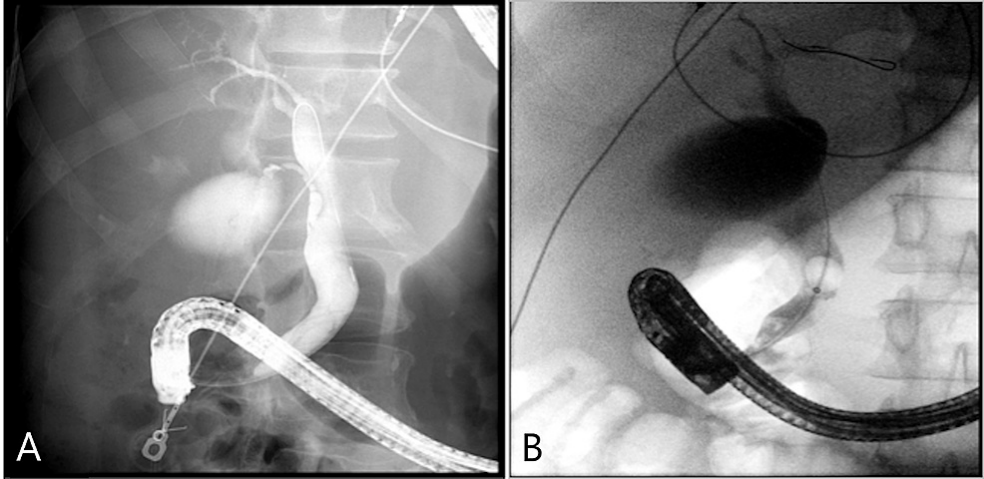
Cholangiogram demonstrating patent cystic duct (A) and filling defects noted on balloon sweep (B)

**Figure 3:**
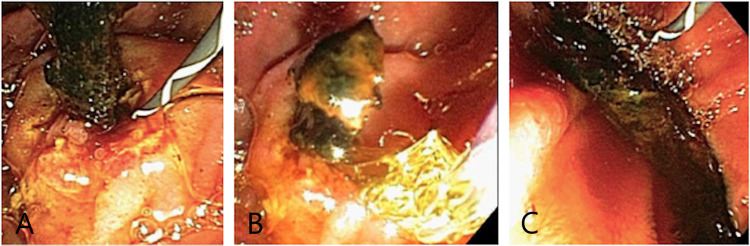
Bile duct stone cast in a transpapillary position post sphincterotomy and attempted extraction (A); status post sphincteroplasty (B); stone successfully extracted (C)

ERCP confirmed a distal linear filling defect within the lower CBD (Figure 2). Interestingly, there was CBD dilation without intrahepatic duct dilation (Figure 3). A sphincterotomy and balloon sphincteroplasty were performed. The linear bile duct stone in the shape of a bile duct cast was extracted using balloon sweep. There was mild oozing from his sphincterotomy, and hence his bile duct was stented given the need for the resumption of anticoagulation. A bile duct aspirate grew *Staphylococcus epidermidis*. After the procedure, his condition was found to be good with improvement in his liver enzymes. His antibiotics were deescalated to oral medications and his diet was advanced. His acute cholecystitis was managed with antibiotics. General surgery reviewed his case and offered an outpatient cholecystectomy.

**Figure 4:**
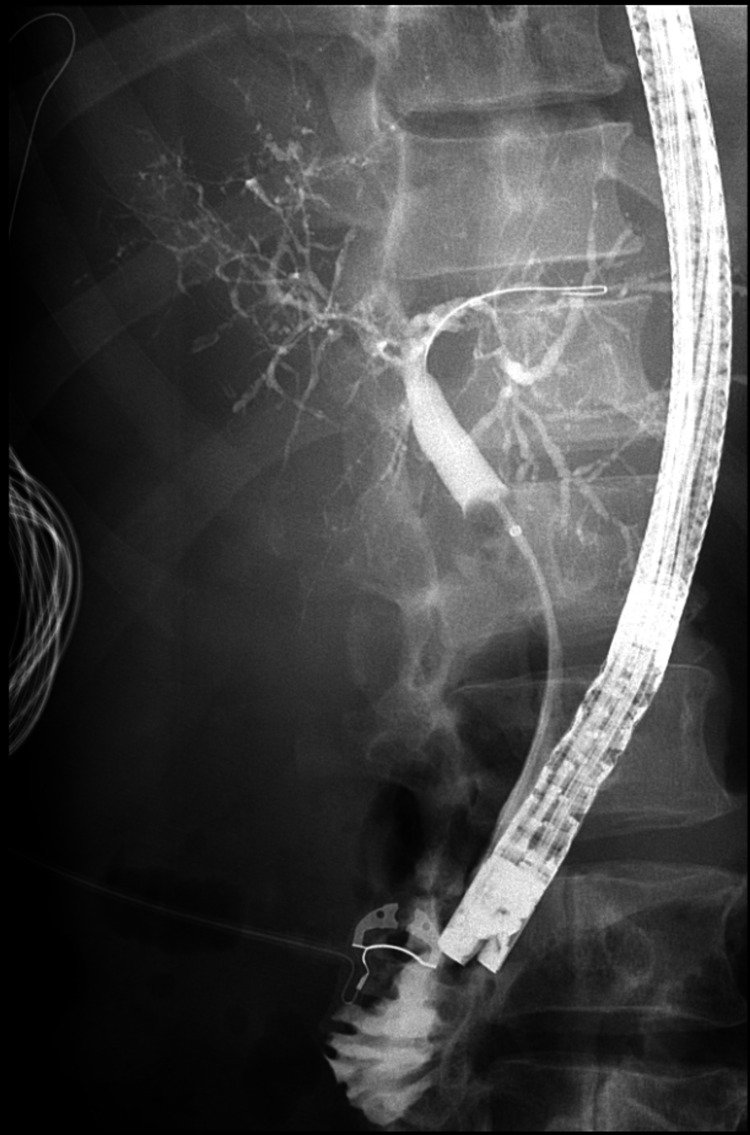
Intrahepatic duct stenosis without dilation, consistent with secondary sclerosing cholangitis

A repeat outpatient ERCP was performed for stent removal. During this exam, his stents and further bile duct casts were removed. However, the second ERCP was indicative of significant intrahepatic ductal disease (Figure 4). He was evaluated by hepatology and diagnosed with SSC-CIP. Primary sclerosing cholangitis, congestive hepatopathy, and other causes of secondary sclerosing cholangitis were excluded.

## Discussion

This case is interesting for several reasons. Three weeks prior to the patient’s presentation with cholangitis, his mother had been diagnosed with acute calculous cholecystitis requiring an urgent cholecystectomy. His daughter has developed biliary colic and is pending consultation with general surgery. All three family members had COVID-19 at the same time. However, only the patient described here was hospitalized for COVID-19 and neither of the other two family members had COVID-19 pneumonia or liver disease. Independent of his ICU stay with COVID-19, the patient's risk factors for stone formation included significant weight loss, TPN, and Hispanic ethnicity.

Cases of acalculous cholecystitis in the context of COVID-19 have been reported in the literature [5]. One case report has also described secondary sclerosing cholangitis in a critically ill patient precipitated by COVID-19 [6]. It is postulated that SSC-CIP may be secondary to both bile duct ischemia and altered bile composition [7,8]. Bile duct epithelium is supplied by the hepatic arterial branches and the delicate peribiliary plexus [9]. Bile duct ischemia leads to canaliculicular dysfunction and cholestasis [10]. As described in a previous retrospective review, our case demonstrated biliary casts and pruning of the biliary tree, without any damage to the CBD [11]. What is unique in this case is that the patient's biliary casts caused cholangitis and his SSC-CIP was diagnosed on ERCP and not magnetic resonance cholangiopancreatography (MRCP) [11]. If ERCP is performed for SSC-CIP, three patterns can be seen on the cholangiogram. The first, as seen in this patient, is biliary casts. Bile duct casts are characteristic of SSC-CIP and are present in up to 87% of cases. The second is the loss of intrahepatic ducts secondary to destruction. The final pattern is a significant small duct drop-out with the cholangiogram resembling a pruned tree [11]. 

The standard management for bile duct casts is sphincterotomy and extraction [12]. Intrahepatic bile duct destruction and pruning are not amenable to endoscopic therapy. The prognosis of SSC-CIP is dependent on coexisting liver disease and secondary liver injury. This patient's lack of intrahepatic duct dilation was concerning for advanced liver fibrosis secondary to SSC-CIP. Unfortunately, once the progression from SSC-CIP to cirrhosis occurs, it becomes irreversible. The definitive management method is liver transplant [13].

## Conclusions

SSC-CIP is a significant complication of an ICU stay. Severe COVID-19 is a multisystem disease associated with a cascade of complications. Unfortunately, beyond cholestasis, this case involved the serious complication of SSC-CIP from COVID-19. The case was further complicated by acute cholangitis from obstructive bile duct casts.
